# Role of SIRT3 in the regulation of *Gadd45α* expression and DNA repair in β-cells

**DOI:** 10.1016/j.jbc.2025.108451

**Published:** 2025-03-25

**Authors:** Aaron Naatz, Kelsey S. Bohl, Rachel A. Jones Lipinski, Joshua A. Nord, Alyssa L. Gehant, Polly A. Hansen, Brian C. Smith, John A. Corbett

**Affiliations:** Department of Biochemistry, Medical College of Wisconsin, Milwaukee, Wisconsin, USA

**Keywords:** β-cell, nitric oxide, sirtuin, Sirt3, Gadd45α, DNA repair

## Abstract

In previous studies, we have shown that growth arrest and DNA damage (*Gadd*) 45α is required for the repair of nitric oxide-mediated DNA damage in **β**-cells. *Gadd45α* expression is stimulated by nitric oxide and requires forkhead box protein (Fox) O1 and NAD^+^-dependent deacetylase activity. Based on inhibitor studies, we attributed this activity to Sirtuin (SIRT)1; however, the inhibitors used in this previous study also attenuate the deacetylase activity of SIRT2, 3, and 6. We now provide experimental evidence that SIRT1 is dispensable for **β**-cell expression of *Gadd45**α*** and that the mitochondrial localized isoform SIRT3, is required for DNA repair in **β**-cells. We show that siRNA knockdown of *Sirt3* attenuates nitric oxide-stimulated *Gadd45**α*** mRNA accumulation in both wildtype and *Sirt1*^*−/−*^ INS 832/13 cells as well as isolated rat islets and that SIRT3 inhibition increases FoxO1 acetylation and attenuates DNA repair in response to nitric oxide. While SIRT3 is predominantly localized to mitochondria, a small fraction is localized in the nucleus of insulin-containing cells and functions to participate in the regulation of FoxO1-dependent, nitric oxide-stimulated DNA repair.

Insulin-dependent type 1 diabetes (T1D) is an autoimmune disease characterized by the selective destruction of insulin-secreting β-cells (1, 2). While T-lymphocytes play a primary role in mediating the loss of β-cell mass during disease development (2), many studies have suggested that proinflammatory cytokines, such as interleukin (IL)-1, contribute to disease development by inhibiting insulin secretion and stimulating a loss in functional β-cell mass (3, 4, 5, 6). In support of a role in diabetes development, IL-1 inhibits insulin secretion in a time-dependent manner that is first apparent ∼5 to 8 h after cytokine addition, and complete following an 18 h incubation (3, 7). The inhibitory actions of IL-1 require new gene expression and protein synthesis (7), and correlate with an inhibition of mitochondrial oxidative metabolism (8, 9). The mechanism by which IL-1 inhibits insulin secretion is well-known and has been extensively characterized (10). Cytokines stimulate the expression of the inducible isoform of nitric oxide synthase (iNOS) in endocrine cells (11, 12). iNOS produces low micromolar levels of nitric oxide that inhibit the Krebs Cycle enzyme aconitase by destroying the iron-sulfur clusters (13, 14) and the electron transport chain (ETC) by competing with oxygen (terminal electron acceptor) for binding to complex 4 (15). The inhibition of mitochondrial oxidative metabolism is the mechanism by which cytokines inhibit insulin secretion (8, 9, 11, 13).

Cytokines have been proposed to be toxic to β-cells, either by necrosis or apoptosis, and the role of nitric oxide in this process has been controversial as some studies suggest that cytokines induce β-cell apoptosis in a nitric oxide-independent manner (16, 17, 18, 19). In contrast to this view, the combination of IL-1, TNF, and IFN-γ fails to decrease the viability or apoptosis of islet cells isolated from mice lacking iNOS (20). Also, mice expressing iNOS under the control of the insulin promoter (β-cell selective expression) develop nitric oxide-dependent diabetes (21). The type of cell death induced by cytokines has also been controversial (18, 19, 22). Although some studies suggest that β-cells die by apoptosis, it is challenging to observe more than a 2-fold increase in caspase activity in primary islet cells in response to cytokine treatment (16). DNA binding dyes have been used as indicators of apoptosis, but these approaches lack specificity for the type of cell death. Also, nitric oxide is known to induce DNA damage including DNA strand breaks that are detectable by TUNEL staining (23), resulting in activation of the DNA damage response (DDR), as evidenced by phosphorylation of H2AX (24). While TUNEL positivity has been equated with apoptosis, this assay detects DNA strand breaks and is not specific for apoptosis. Furthermore, we have shown that nitric oxide, when produced at micromolar levels by iNOS, is an effective inhibitor of caspase activation and apoptosis in insulinoma cells following treatment with the apoptosis inducers tunicamycin (25) and camptothecin (26).

Pancreatic β-cells are unique in the mechanisms controlling oxidative metabolism. Glut2 and glucokinase sense extracellular glucose levels, but it is the rate of mitochondrial oxidative metabolism that determine the amount of insulin secreted. This is possible because glycolysis and mitochondrial oxidation are coupled such that 90% of the carbons of glucose that enter β-cells are oxidized to CO_2_ in a concentration-dependent manner (27, 28, 29). In almost all other cell types, glycolysis and mitochondrial oxidative metabolism are uncoupled. This allows cells the metabolic flexibility to continue glycolysis when oxygen is limited due to the regeneration of NAD^+^ by lactate dehydrogenase for use by glyceraldehyde 3-phosphate dehydrogenase activity (27, 28, 29). Thus, when mitochondrial oxidative metabolism is impaired, most cells maintain ATP levels *via* glycolysis while β-cells lack this metabolic flexibility (30). Since most cell viability assays are measures of metabolism, ATP, NAD^+^, or mitochondrial membrane potential, it is not surprising that cytokines are believed to be toxic to β-cells as nitric oxide is a potent inhibitor of oxidative metabolism.

If cell viability is lost, either by apoptosis or necrosis, then one would expect that the actions of cytokines would be irreversible; yet in rat, mouse, and human islets cytokine-mediated damage is completely reversible. Comens *et al.* (31) first showed that the inhibitory actions of a 15 h incubation with IL-1 on insulin secretion are completely reversible if the cytokine is removed by washing and the islets are cultured in the absence of IL-1 for four additional days. The recovery window can be reduced from 4 days to 8 h by adding an inhibitor of iNOS to the culture (in the presence of the cytokine) (32, 33). Only when islets are cultured for extended periods of time greater than 36 h are the actions of cytokines irreversible (33) and under these conditions, ATP and NAD^+^/NADH are depleted (30). In many cases, DNA damage has been used to indicate cell death; however, β-cells maintain robust mechanisms to repair this damage (34, 35). We have shown that base excision repair (BER) is the pathway used by β-cells to repair damaged DNA and that this pathway requires FoxO1-dependent expression of growth arrest and DNA damage-inducible 45 (*Gadd45)α* (34, 35). The activity of FoxO1 can be regulated by deacetylases, specifically silent mating type information regulation two homolog (SIRT) or sirtuins (36, 37, 38). Using isoform-selective inhibitors, we showed that SIRT1, a member of this NAD^+^-dependent sirtuin deacetylase family was required for FoxO1-dependent *Gadd45α* expression; however, there is broad overlap in the function of the seven members (SIRT1-7) of the sirtuin protein family (39), and isoform-selective inhibitors can be effective at attenuating the activity of multiple sirtuin isoforms (40). These issues raise the possibility that isoforms other than SIRT1 may regulate the repair of damaged β-cell DNA in response to nitric oxide. Consistent with that hypothesis, we now show that nitric oxide stimulates the expression of *Gadd45α* in insulinoma cells lacking SIRT1. Using biochemical and molecular approaches we provide evidence that SIRT3, historically believed to be a mitochondrial localized member of this family, plays a primary role in regulating β-cell expression of *Gadd45α* and DNA repair in β-cells exposed to nitric oxide.

## Results

### Regulation of nitric oxide-dependent *Gadd45**α*** mRNA expression in **β**-cells by FoxO1 and sirtuins

**Figure 1:**
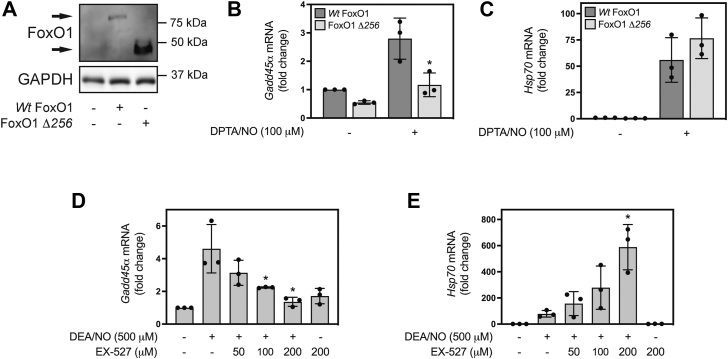
**FoxO1 and sirtuin activity are required for nitric oxide-stimulated *Gadd45α* mRNA accumulation in INS 832/13 cells.** INS 832/13 cells were transduced with adenoviral constructs containing either wild-type FoxO1 or FoxO1 Δ*256* (*A*–*C*). Following a 24 h culture, FoxO1 construct expression and GAPDH (loading control) were determined by Western blot analysis (*A*), or cells were treated with 100 μM DPTA/NO for 3 h and *Gadd45α* and *Hsp70* mRNA accumulation was measured by RT qPCR (*B* and *C*). INS 832/13 cells were pretreated with indicated concentrations of EX-527 for 30 min, followed by a 3 h treatment with 500 μM DEA/NO, and *Gadd45α* and *Hsp70* mRNA accumulation was determined by RT qPCR (*D* and *E*). Results are representative (*A*) or the average ± SD (*B*–*E*) of three independent experiments. Statistically significant changes relative to DPTA/NO or DEA/NO treatment (∗*p* < 0.05).

When produced at iNOS-derived micromolar levels, nitric oxide induces DNA damage in β-cells (22, 41). At the same time, nitric oxide also activates DNA repair pathways including the expression of *Gadd45α* and BER of damaged DNA (34). Several years ago, we showed that FoxO1 and SIRT1 activity are required for nitric oxide-stimulated *Gadd45α* expression and DNA repair in β-cells (35), and consistent with these findings, DPTA/NO-stimulated *Gadd45α* mRNA accumulation is attenuated in INS 832/13 cells transduced with an adenoviral vector expressing a truncated dominant negative mutant of FoxO1 lacking the transactivation domain (FoxO1 Δ*256*) (Fig. 1, *A* and *B*) (42). Transduction with an adenovirus expressing wild-type FoxO1 did not modify DPTA/NO-stimulated *Gadd45α* mRNA accumulation in INS 832/13 cells (Fig. 1*B*). The inhibitory actions of the dominant negative FoxO1 mutant appear selective for *Gadd45α* expression as FoxO1 Δ*256* did not modify *Hsp70* mRNA accumulation in response to nitric oxide treatment (Fig. 1*C*).

The regulation of gene expression by FoxO1 can be modified by the activity of members of the sirtuin family of NAD^+^-dependent deacetylases, where nuclear acetylated FoxO1 promotes the expression of apoptosis-related genes, while nuclear deacetylated FoxO1 promotes the expression of DNA repair and stress resistance genes (43, 44). Consistent with our previous findings (35), pretreatment of INS 832/13 cells for 30 min with increasing concentrations of the SIRT1 inhibitor, EX-527 (50, 100, and 200 μM) attenuates nitric oxide-stimulated *Gadd45α* mRNA accumulation (3 h treatment with 500 μM DEA/NO) without inhibiting DEA/NO-stimulated *Hsp70* mRNA accumulation (Fig. 1, *D* and *E*). In fact, this SIRT1 inhibitor enhanced nitric oxide-stimulated *Hsp70* mRNA in a concentration-dependent manner (Fig. 1*E*).

### The role of SIRT1 in the **β**-cell response to nitric oxide and gene expression

**Figure 2:**
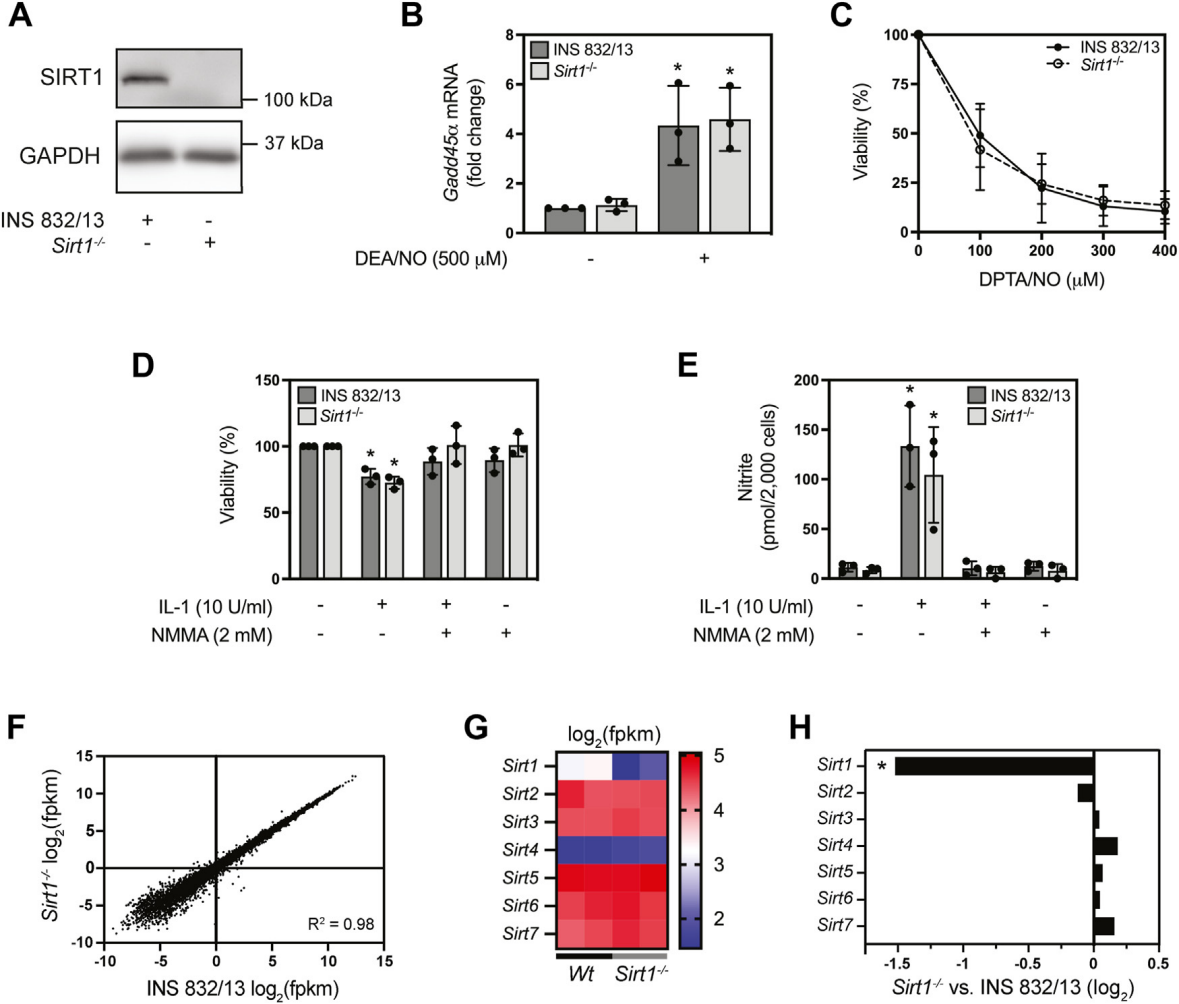
**Efects of nitric oxide in *Sirt1*-deficient INS 832/13 cells.***Sirt1* was deleted from INS 832/13 cells by CRISPR/Cas9 and protein loss was confirmed by Western blot analysis with GAPDH serving as a loading control (*A*). *Gadd45α* mRNA accumulation in response to a 3 h incubation with DEA/NO (500 μM) was determined by RT qPCR in wild-type and *Sirt1*^*−/−*^ INS 832/13 cells (*B*). The concentration-dependent effects of DPTA/NO on wild-type and *Sirt1*^−/−^ INS 832/13 cell viability were measured using the neutral *red* assay (*C*). INS 832/13 and *Sirt1*^*−/−*^ INS 832/13 were treated with IL-1 (10 U/ml) in the presence or absence of the NOS inhibitor NMMA (2 mM) for 24 h and cell viability was determined using the neutral *red* assay (*D*) and nitrite production was determined by Griess assay on the culture supernatant (*E*). Steady-state mRNA levels were examined by RNAseq comparing INS 832/13 and *Sirt1*^*−/−*^ INS 832/13 cells (*F*–*H*). Changes in overall mRNA expression (log_2_ fpkm) were determined by differential expression analysis (*F*). Differences in sirtuin isoform mRNA expression in *Sirt1*^*−/−*^ INS 832/13 relative to wild-type cells are shown by heat map (log_2_ fpkm) (*G*) or fold change (log_2_) (*H*). Results are representative of three (*A*), the average ± SD of three (*B*–*E*), or the average of two (*F*–*H*) independent experiments. Statistically significant changes relative to untreated controls are indicated (∗*p* < 0.05).

To confirm that the target of EX-527 is SIRT1, the CRSPR-Cas9n ‘double nickase’ method was used to generate INS 832/13 cells lacking this sirtuin (45). Although SIRT1 protein was not detected in the *Sirt1*^*−/−*^ INS 832/13 cell line (Fig. 2*A*), nitric oxide stimulated the accumulation of *Gadd45α* mRNA to similar levels in the wild-type and *Sirt1*^*−/−*^ INS 832/13 cells (Fig. 2*B*). Further, the loss of INS 832/13 cell viability (neutral red assay, which provides an indirect measure of cellular ATP) in response to nitric oxide (DPTA/NO) (Fig. 2*C*) or treatment with IL-1 was not modified when comparing wild-type and *Sirt1*^*−/−*^ INS 832/13 cells; however, the iNOS inhibitor N^G^-monomethyl L-arginine (NMMA) attenuated the loss of INS 832/13 cell viability following a 24 h treatment with IL-1 (Fig. 2*D*). Nitrite (oxidative product of nitric oxide) levels are increased in response to IL-1 and attenuated by the NOS inhibitor NMMA in both cell types (Fig. 2*E*). Because the responses to nitric oxide and IL-1 were not modified in *Sirt1*^*−/−*^ INS 832/13 cells, RNA sequencing (RNAseq) of wild-type and *Sirt1*^*−/−*^ INS 832/13 cells was performed to identify potential changes in gene expression associated with the loss of SIRT1 (Fig. 2, *F*–*H*). Overall, there were few changes in gene expression between the two cell lines when comparing log_2_ (fpkm) values (Fig. 2*F*) and there does not appear to be compensation for the loss of SIRT1 by other sirtuin family members (Fig. 2, *G* and *H*). These surprising results suggest that SIRT1 is not required for nitric oxide-stimulated *Gadd45α* mRNA accumulation in β-cells and does not appear to be involved in the larger β-cell response to nitric oxide or IL-1.

### Isoform selectivity of the sirtuin inhibitor EX-527

**Figure 3:**
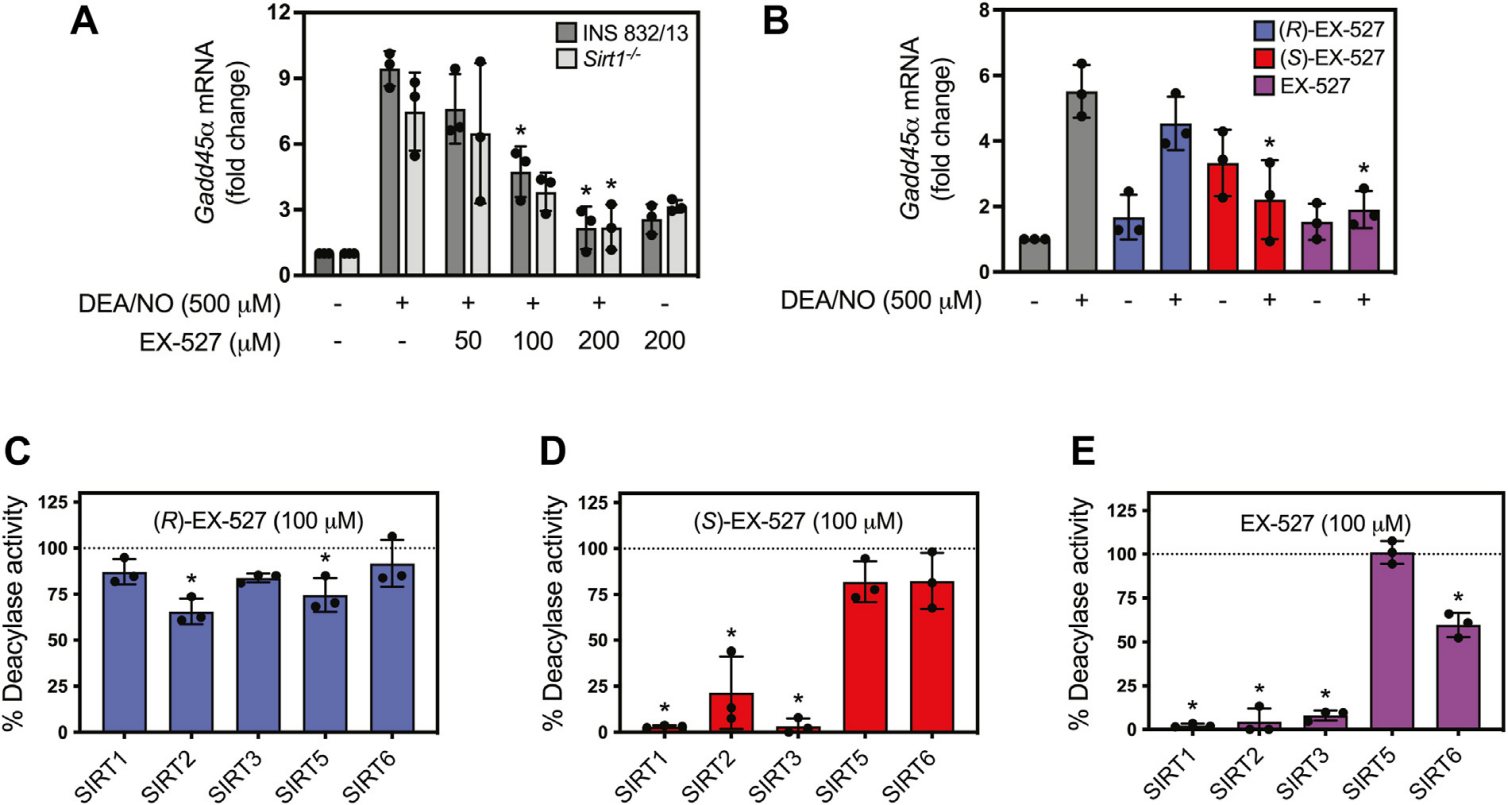
**Effects of EX-527 on nitric oxide-stimulated *Gadd45α* mRNA accumulation in INS 832/13 cells and the deacetylase activity of sirtuin isoforms.** INS 832/13 and *Sirt1*^*−/−*^ INS 832/13 cells were pretreated for 30 min with indicated concentrations of EX-527 (*A*), 100 μM (*R*)- or (*S*)-EX-527 enantiomers or 200 μM EX-527 racemate (*B*), DEA/NO (500 μM) was added and the cells were cultured for 3 h. The cells were harvested and *Gadd45α* mRNA accumulation was determined by RT qPCR (*A* and *B*). The effects of 100 μM (*R*)- or (*S*)-enantiomers and EX-527 racemate on the deacetylase activity of indicated human recombinant sirtuin enzymes were determined and displayed relative to the untreated control (*dotted line*, *C*–*E*). Results are the average ± SD of three independent experiments (*A*–*E*). Statistically significant changes relative to DEA/NO (*A* and *B*) or untreated control activity (*C*–*E*) are indicated (∗*p* < 0.05).

Although EX-527 is described as a selective SIRT1 inhibitor, it attenuates nitric oxide-stimulated *Gadd45α* mRNA accumulation in wild-type and *Sirt1*^*−/−*^ INS 832/13 cells (Fig. 3*A*). These findings are consistent with a lack of selectivity and suggest that it may target several sirtuin isoforms or have additional off-target actions (46, 47, 48). EX-527 is produced in a racemic mixture of the active inhibitor (*S*)-enantiomer and the inactive (*R*)-enantiomer (46, 49). We first examined the effects of the racemic mixture and each enantiomer on nitric oxide-stimulated *Gadd45α* mRNA accumulation in INS 832/13 cells. In a similar concentration-dependent manner, EX-527 attenuated *Gadd45α* mRNA accumulation in response to nitric oxide in wild-type and *Sirt1*^*−/−*^ INS 832/13 cells (Fig. 3*A*). At 200 μM, EX-527 maximally inhibited nitric oxide-stimulated *Gadd45α* mRNA accumulation as did 100 μM of the active (*S*)-enantiomer of EX-527, while the inactive (*R*)-enantiomer of EX-527 did not modify nitric oxide stimulated *Gadd45α* mRNA accumulation in INS 832/13 cells (Fig. 3*B*). Because EX-527 inhibits nitric oxide stimulated *Gadd45α* mRNA accumulation in wild-type and INS 832/13 cells lacking SIRT1, the isoform specificity of this inhibitor was evaluated. Both the racemic mixture and (*S*)-enantiomer of EX-527 are potent inhibitors of SIRT1, SIRT2 and SIRT3 deacetylase activity. The racemic mixture of EX-527 also inhibited SIRT6 deacetylase activity by ∼40% at a concentration of 100 μM (Fig. 3, *D* and *E*). These findings indicate that the inhibitory effects of EX-527 on nitric oxide-dependent *Gadd45α* mRNA accumulation may be mediated by inhibiting the deacetylase activity of SIRT2, SIRT3, or SIRT6.

### The role of SIRT2 in nitric oxide-dependent *Gadd45**α*** mRNA expression in **β**-cells

**Figure 4:**
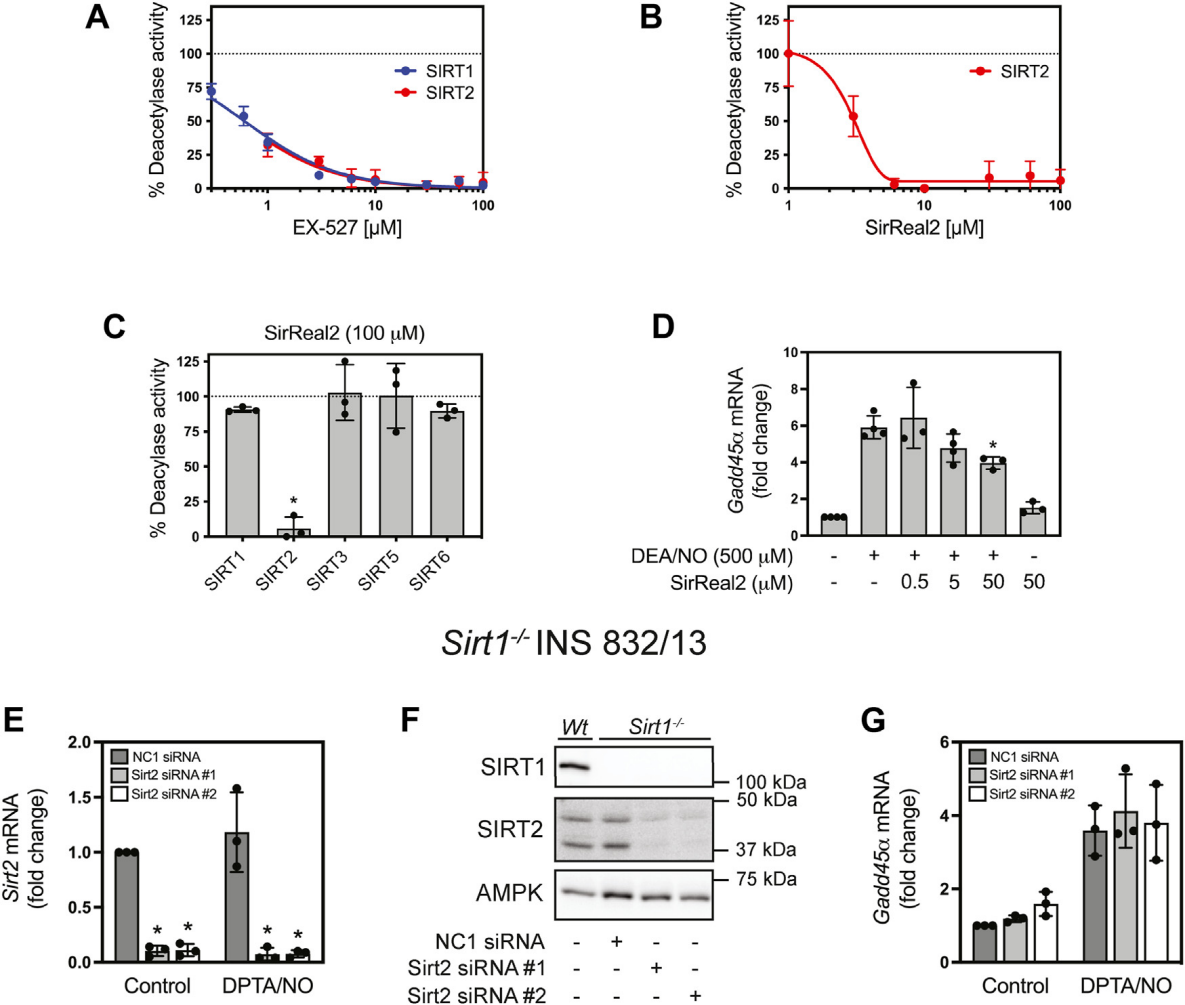
**Role of SIRT2 in regulating nitric oxide-dependent *Gadd45α* mRNA accumulation in INS 832/13 cells.** The concentration-dependent effects of EX-527 on human recombinant SIRT1 and SIRT2 deacetylase activity (*A*) and the concentration-dependent effects of the SIRT2 selective inhibitor, SirReal2, on SIRT2 deacetylase activity were determined (*B*). The effects of SirReal2 (100 μM) on the deacetylase activity of indicated human recombinant sirtuin isoforms were determined (*C*). The activity of the untreated control is indicated by the dotted line (*A*–*C*). INS 832/13 cells were preincubated for 30 min with the indicated concentrations of SirReal2, DEA/NO (500 μM) was added, and following a 3 h incubation the cells were harvested, and *Gadd45α* mRNA accumulation was measured by RT qPCR (*D*). Two independent siRNAs were used to deplete *Sirt2* mRNA from *Sirt1*^*−/−*^ INS 832/13 as determined by RT qPCR (*E*). SIRT1 and SIRT2 protein levels were evaluated by Western blot analysis to confirm depletion in *Sirt1*^*−/−*^ INS 832/13, and AMPK is shown as a loading control (*F*). *Gadd45α* mRNA accumulation in response to 3 h treatment with DPTA/NO (100 μM) was determined by RT qPCR (*G*). Results are the average ± SD (*A*–*E*, and *G*) or representative (*F*) of three to four independent experiments. Statistically significant changes relative to untreated controls (*C* and *E*) or compared to DEA/NO (*D*) are indicated (∗*p* < 0.05). There were no statistically significant changes in *Gadd45α* mRNA accumulation relative to negative control (NC1) siRNA-transfected cells treated with DPTA/NO following depletion of SIRT1 and SIRT2 (*G*).

We initiated our studies by examining SIRT2 because it has been shown to deacetylate FoxO1 (50) and has been found in the nucleus (51). As shown in Figure 4, EX-527 (0.3–100 μM) inhibited the deacetylase activity of SIRT1 and SIRT2 in a concentration-dependent manner with nearly identical kinetics. Unlike EX-527, SirReal2 (52) is a chemically distinct inhibitor of SIRT2 deacetylase activity at concentrations <10 μM (Fig. 4*B*) and it is selective for SIRT2 over other sirtuin isoforms (Fig. 4*C*). SirReal2 did not prevent *Gadd45α* mRNA accumulation in response to nitric oxide when used at concentrations up to 50 μM (Fig. 4*D*). These findings suggest that SIRT2 does not mediate the stimulatory actions of nitric oxide on *Gadd45α* expression; however, the functions of SIRT1 and SIRT2 could be redundant. To address this possibility, siRNA was used to knock down *Sirt2* mRNA in the *Sirt1*^*−/−*^ INS 832/13 cells (Fig. 4*E*). While this resulted in a >90% knockdown of Sirt2 (Fig. 4, *E* and *F*), it did not modify the stimulatory effects of nitric oxide on *Gadd45α* mRNA accumulation (Fig. 4*G*).

### SIRT6 and SIRT7 do not participate in the regulation of *Gadd45**α*** expression in response to nitric oxide

**Figure 5:**
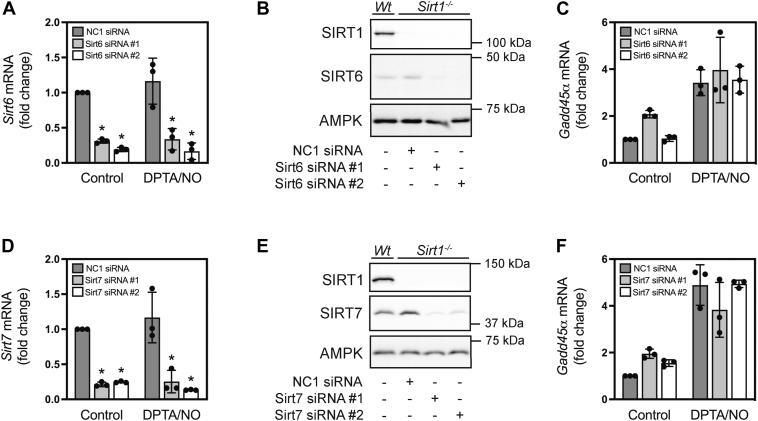
**Effects of SIRT6 and SIRT7 depletion on nitric oxide-stimulated *Gadd45α* mRNA accumulation in INS 832/13 cells.** Two different siRNAs targeting *Sirt6* (*A*–*C*) and *Sirt7* (*D*–*F*) were transfected into *Sirt1*^*−/−*^ INS 832/13 cells and loss of the targeted sirtuin was evaluated by RTqPCR (*A* and *D*). SIRT1, SIRT6, and SIRT7 protein levels were evaluated by Western blot analysis to confirm depletion in *Sirt1*^*−/−*^ INS 832/13, and AMPK is shown as a loading control (*B* and *E*). The effect of SIRT6 (*C*) and SIRT7 (*F*) depletion on DPTA/NO (100 μM) stimulated *Gadd45α* mRNA accumulation following a 3 h incubation was determined by RT qPCR. Results are the average ± SD (*A*, *C*, *D*, and *F*) or representative (*B* and *E*) of three independent experiments. There were no statistically significant changes in *Gadd45α* mRNA accumulation relative to negative control (NC1) siRNA-transfected cells treated with DPTA/NO following depletion of SIRT1 and SIRT6 or SIRT7 (*C* and *F*). Statistically significant changes in *Sirt6* and *Sirt7* mRNA levels relative to the untreated control (*A* and *D*) are indicated (∗*p* < 0.05).

SIRT6 has been reported to participate in BER of DNA that has been damaged in response to oxidants (53) and EX-527 (100 μM) inhibits its deacetylase activity by ∼40% (Fig. 3*E*). siRNA was used to deplete SIRT6 in *Sirt1*^*−/−*^ INS 832/13 cells (Sirt6 expression was decreased by >90%) (Fig. 5, *A* and *B*), yet this decrease in SIRT6 did not modify *Gadd45α* mRNA accumulation in response to a 3 h treatment with nitric oxide (Fig. 5*C*). SIRT7 is a nuclear-localized isoform (54) that has been shown to have regulatory roles in glucose and lipid homeostasis (38, 55) in addition to its role in DNA repair (56). Using siRNA, greater than 90% of Sirt7 was depleted in *Sirt1*^*−/−*^ INS 832/13 cells (Fig. 5, *D* and *E*), and much like the other sirtuin isoforms (1, 2, and 6), SIRT7 depletion did not alter *Gadd45α* mRNA accumulation in response to 3 h incubation with nitric oxide (Fig. 5*F*). Much like SIRT1 and SIRT2, these findings indicate that neither SIRT6 nor SIRT7 participate in the regulation of *Gadd45α* expression in nitric oxide treated β-cells.

### The role of SIRT3 in nitric oxide-dependent *Gadd45***α**** mRNA expression in **β**-cells

**Figure 6:**
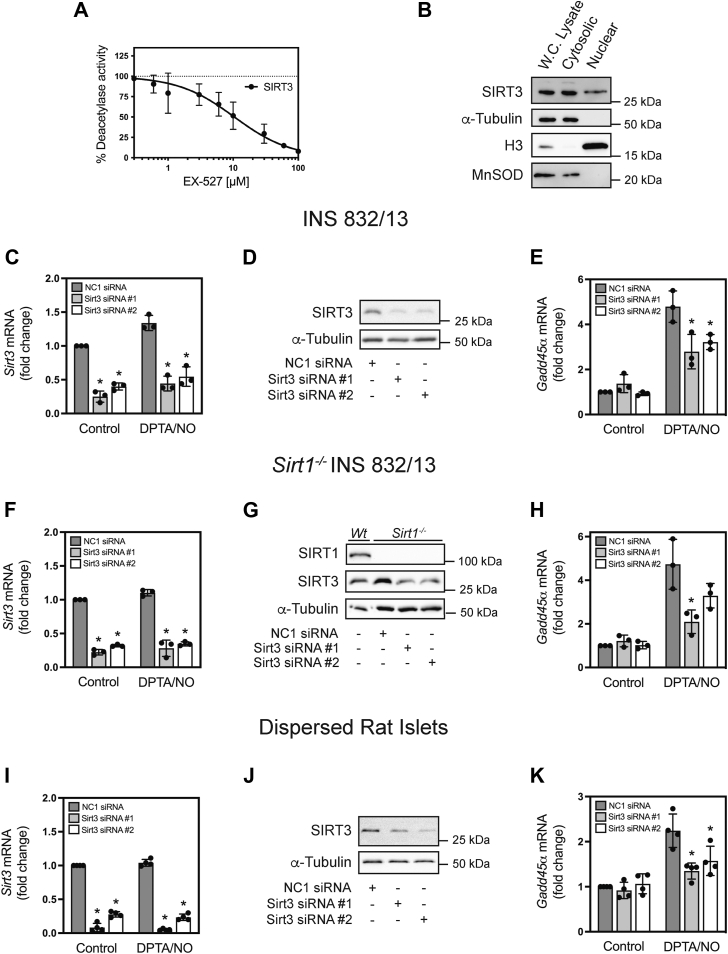
**Effects of SIRT3 depletion on nitric oxide-stimulated *Gadd45α* mRNA accumulation in INS 832/13 and dispersed rat islet cells.** The concentration-dependent effects of EX-527 on SIRT3 deacetylase activity were examined with untreated control deacetylase activity indicated by the *dotted line* (*A*). SIRT3 cellular localization was determined using cell fractionation of whole cell lysates into cytosolic (containing mitochondria) and nuclear fractions followed by Western blot analysis of INS 832/13. Fraction markers include α-tubulin for the cytoplasm marker, histone H3 for the nucleus, and MnSOD for the mitochondria (*B*). INS 832/13 cells (*C*–*E*), *Sirt1*^*−/−*^ INS 832/13 cells (*F*–*H*), and dispersed rat islet cells (*I*–*K*) were depleted of *Sirt3* following transfection of two different siRNAs, and steady-state levels of *Sirt3* mRNA were determined by RT qPCR (*C*, *F*, and *I*). SIRT3 depletion (*D*, *G*, and *J*) and SIRT1 depletion (*G*) were determined by Western blot analysis with α-tubulin used as a loading control (*D*, *G*, and *J*). The effects of SIRT3 depletion on DPTA/NO (100 μM) stimulated *Gadd45α* mRNA accumulation following a 3 h incubation were determined by RT qPCR using INS 832/13 cells (*E*), *Sirt1*^*−/−*^ INS 832/13 cells (*H*), or dispersed rat islet cells (*K*). Results are the average ± SD (*A*, *C*, *E*, *F*, *H*, *I*, and *K*) or representative (*B*, *D*, *G*, and *J*) of three to four independent experiments. Statistically significant changes in *Gadd45α* mRNA accumulation relative to the negative control (NC1) siRNA-transfected cells treated with DPTA/NO (*E*, *H*, and *K*) and Sirt3 mRNA levels relative to NC1 (*C*, *F*, and *I*) are indicated (∗*p* < 0.05).

We initiated our studies by examining sirtuin isoforms that we believed would be likely regulators of FoxO1-dependent *Gadd45α* expression and DNA repair in β-cells based on inhibitor sensitivity and cellular localization. SIRT1, 2, 6, and seven are localized to the nucleus where they regulate gene expression. We did not initially explore SIRT3 because this isoform is predominantly localized to the mitochondria and has been shown to participate in the regulation of mitochondrial oxidative metabolism (38). However, several reports have suggested that SIRT3 can be found in the nucleus and that it can translocate between the mitochondria and the nucleus (57, 58, 59). As shown in Figure 6*A*, EX-527 inhibited SIRT3 deacetylase activity in a concentration-dependent manner with maximal inhibition at 100 μM. To determine the cellular localization of SIRT3, INS 832/13 cells were fractionated into cytosolic (containing mitochondria) and nuclear compartments (Fig. 6*B*) followed by Western blot analysis for SIRT3. As expected, SIRT3 was found in the mitochondria-containing cytosolic fraction along with cytosolic protein marker α-Tubulin and mitochondrial marker MnSOD (Fig. 6*B*). We did not observe the presence of the nuclear marker Histone H3 in the cytosolic fraction. In addition to being mitochondrial, we also observed SIRT3 in the nuclear fraction along with Histone H3 (Fig. 6*B*) indicating that a small fraction of SIRT3 is nuclear in INS 832/13 cells.

Since SIRT3 is found in the nucleus in addition to the mitochondria and its deacetylase activity is sensitive to EX-527, we examined whether siRNA depletion would modify nitric oxide-stimulated *Gadd45α* mRNA accumulation by INS 832/13 cells. Two different siRNAs that target Sirt3 effectively depleted this sirtuin isoform in INS 832/13 cells (Fig. 6, *C* and *D*), INS 832/13 cells deficient in SIRT1 (Fig. 6, *F* and *G*), and primary rat islet cells (Fig. 6, *I* and *J*). The depletion included ∼70% or greater knockdown of *Sirt3* mRNA (Fig. 6, *C*, *F*, and *I*) and SIRT3 protein (Fig. 6, *D*, *G*, and *J*) in each cell type. Importantly, siRNA knockdown of SIRT3 in wild-type and *Sirt1*^*−/−*^ INS 832/13 attenuated the stimulatory actions of nitric oxide on *Gadd45α* mRNA accumulation (Fig. 6, *E* and *H*). The inhibition of Gadd*45α* mRNA accumulation in *Sirt1*-deficient cells depleted of *Sirt3* indicates that SIRT1 and SIRT3 do not serve redundant roles in this pathway. Primary rat islet cells responded much like the INS 832/13 cells where SIRT3 depletion attenuated *Gadd45α* mRNA accumulation in response to nitric oxide (Fig. 6*K*). These findings support SIRT3 as the EX-527 sensitive sirtuin that regulates nitric oxide stimulation of *Gadd45α* mRNA in β-cells.

### Role of SIRT3 in the repair of damaged **β**-cell DNA in response to nitric oxide

**Figure 7:**
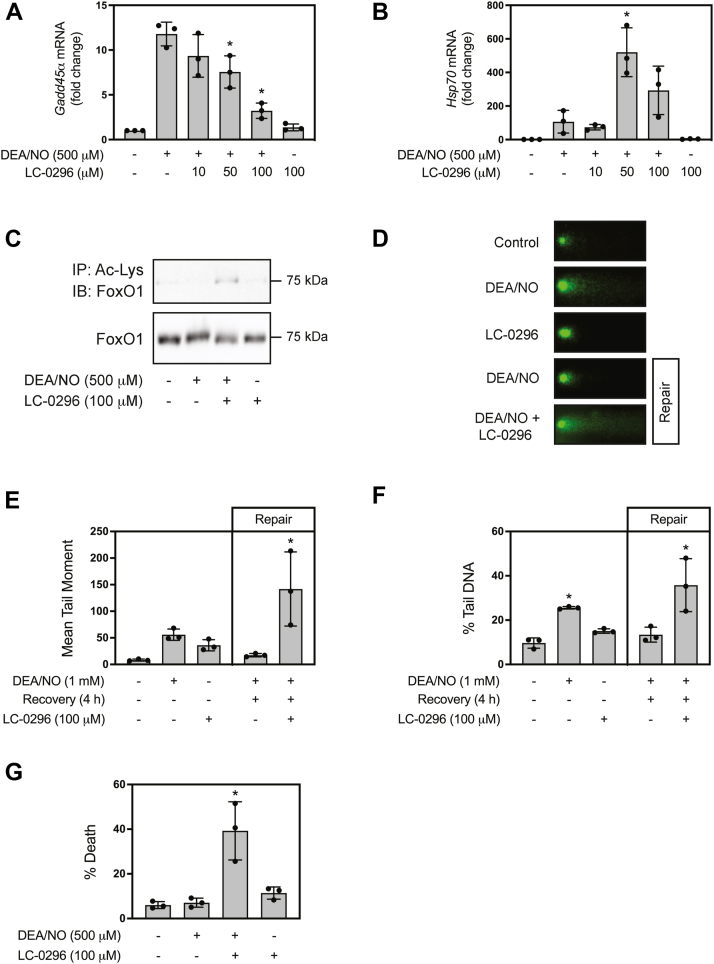
**SIRT3 inhibition attenuates the repair of nitric oxide-mediated DNA damage in INS 832/13 cells.** INS 832/13 were pretreated for 30 min with the indicated concentrations of the SIRT3 selective inhibitor LC-0296 (*A* and *B*), DEA/NO (500 μM) was added, and 3 h later, *Gadd45α* (*A*) and *Hsp70* mRNA (*B*) accumulation were measured by RT qPCR. The acetylation status of FoxO1 was assessed by immunoprecipitating acetylated protein and FoxO1 was identified by Western blot analysis of INS 832/13 cells pretreated for 30 min with LC-0296 followed by 1 h incubation with the indicated concentrations of DEA/NO and LC-0296. Western blot analysis of FoxO1 levels from lysates is shown to control input levels for immunoprecipitation (*C*). DNA damage was evaluated by treating INS 832/13 cells for 1 h with DEA/NO (1 mM) or 5.5 h with LC-0296 (100 μM). The repair of damaged DNA was evaluated by incubating INS 832/13 cells for 1 h with DEA/NO (1 mM) in the presence or absence of LC-0296 (100 μM). The cells were then washed to remove the nitric oxide donor and incubated in the presence or absence of LC-0296 for 4 additional h (*D*–*F*). Fluorescent microscopy was used to characterize damaged DNA by evaluating the comet heads and extended comet tails (*D*) and quantified computationally as a mean tail moment (*E*) and % tail DNA (*F*). The effects of SIRT3 inhibition and nitric oxide on INS 832/13 cell viability were evaluated using SYTOX green staining in cells pretreated for 30 min with LC-0296 (100 μM), followed by the addition of DEA/NO (500 μM) and additional culture for 6 h (*G*). Results are the average ± SD (*A*, *B*, *E*–*G*) or representative (*C* and *D*) of three independent experiments. Statistically significant changes relative to DEA/NO (*A* and *B*) or untreated control (*E*–*G*) are indicated (∗*p* < 0.05).

Knowing that GADD45α participates in the repair of β-cell DNA (34), and that SIRT3 regulates *Gadd45α* mRNA accumulation in response to nitric oxide, the effects of the SIRT3 selective inhibitor, LC-0296 (60), on the repair of damaged DNA in β-cells were examined. As shown in Figure 7*A*, DEA/NO (500 μM)-stimulated *Gadd45α* mRNA accumulation is inhibited in a concentration-related manner by LC-0296 with a maximal effect at 100 μM. The effects of SIRT3 inhibition appear selective, as LC-0296 did not inhibit *Hsp70* mRNA accumulation in response to DEA/NO (Fig. 7*B*). Nitric oxide stimulates FoxO1 nuclear localization, and FoxO1-dependent *Gadd45α* mRNA accumulation (34, 35). Because transcriptional activation by FoxO1 is regulated by its acetylation status and SIRT3 inhibition attenuates nitric oxide-stimulated *Gadd45α* expression, we examined the acetylation status of FoxO1 by immunoprecipitation of acetylated proteins followed by Western blot analysis of FoxO1. As shown in Figure 7*C*, DEA/NO treatment of INS 832/13 cells for 1 h results in a slight decrease in basal levels of FoxO1 acetylation. The SIRT3 inhibitor LC-0296 does not modify basal but increases the level of acetylated FoxO1 in DEA/NO treated cells. These findings provide evidence to support a role for NAD^+^-dependent deacetylation of FoxO1 by SIRT3 in nitric oxide-dependent *Gadd45α* expression by INS 832/13 cells.

The comet assay was used to evaluate the role of SIRT3 in the repair of nitric oxide-damaged DNA in INS 832/13 cells (Fig. 7, *D*–*F*). Treatment with 1 mM DEA/NO for 1 h results in comet formation (Fig. 7*D*) that is quantified as mean tail moment ∼50 (Fig. 7*E*) and as %Tail DNA (Fig. 7*F*). Following this 1 h treatment, the cells were washed to remove nitric oxide and then cultured for 4 h in the absence of nitric oxide to allow for DNA repair. Alone, LC-0296 did not induce DNA damage; however, when present during the incubation with DEA/NO and the 4 h repair incubation, DNA damage was increased >2-fold over the levels observed following a 1 h incubation with DEA/NO alone. Consistent with the accumulation of high levels of damaged DNA, there was a loss in the viability of ∼40% of INS 832/13 cells in response to a 6 h treatment with DEA/NO and LC-0296 as measured by uptake of the DNA binding dye SYTOX green (Fig. 7*G*). Neither DEA/NO nor LC-0296 alone modified the viability of INS 832/13 cells. We interpret these findings to indicate that SIRT3 inhibition attenuates DNA repair and causes the loss of INS 832/13 cell viability in the presence of genotoxic stress induced by nitric oxide. These findings suggest that SIRT3 is the sirtuin isoform that participates in nitric oxide-stimulated *Gadd45α* expression and DNA repair in β-cells.

## Discussion

It is well established that nitric oxide mediates the inhibitory actions of cytokines on insulin secretion and mitochondrial oxidative metabolism and is responsible for the induction of DNA damage (22, 23, 41, 61). While considered damaging, the inhibition of metabolic and secretory function is reversible (32, 33), and β-cells have active pathways to repair damaged DNA (34, 35, 62, 63). In fact, nitric oxide, which is responsible for inducing DNA damage, also activates DNA repair pathways (34, 64). DNA repair in β-cells occurs *via* p53-independent, FoxO1- and Gadd45α-dependent mechanisms (34, 35). FoxO1-dependent transcriptional regulation is controlled, in part, by its acetylation status. Members of the sirtuin family of deacetylases are known to regulate FoxO1-dependent gene expression (43, 44). Using inhibitors originally believed to be isoform-selective, we identified SIRT1 as the sirtuin isoform responsible for regulating nitric oxide-dependent *Gadd45α* expression in β-cells (35); however, several studies have questioned the specificity of isoform-selective inhibitors for their target sirtuin (46, 47, 48). Because of specificity issues, we generated an insulinoma cell line deficient in SIRT1 to further evaluate the role of this sirtuin in regulating *Gadd45α* expression in response to nitric oxide. Surprisingly, the SIRT1 selective inhibitor EX-527 is equally effective at attenuating nitric oxide-stimulated *Gadd45α* expression in both wild-type and *Sirt1*^*−/−*^ INS 832/13 cells (Fig. 3*A*). Further, knocking out *Sirt1* had little effect on the overall gene expression pattern in these cells as evaluated by RNAseq (Fig. 2*F*). These findings suggested that a sirtuin activity other than SIRT1 controls the expression of DNA repair genes in β-cells or that the activity of SIRT1 is redundant with additional isoforms.

To identify the sirtuin isoform involved in this process, we evaluated the effects of EX-527 on the deacetylase activity of purified enzymes of most of the relevant members of this family. After confirming that the inhibitory actions are mediated by the active (*S*)-enantiomer (Fig. 3, *B* and *D*), we showed that EX-527 is a potent inhibitor of SIRT1, SIRT2, SIRT3, and a less effective inhibitor of SIRT6 (∼40% inhibition) (Fig. 3*E*). Genetic approaches (siRNA) were used to knock down each of the sirtuin isoforms in wild-type and *Sirt1*^*−/−*^ INS 832/13 cells. Initial studies focused on SIRT6 because it is nuclear localized and has been shown to function in BER of damaged DNA (53), and SIRT2 because it can deacetylate FoxO1 (50) and has been shown to translocate to the nucleus (51). Overall, depletion of SIRT2 or SIRT6 did not modify nitric oxide-stimulated *Gadd45α* expression in *Sirt1*^*−/−*^ INS 832/13 cells (Figs. 4*G* and 5*C*). The lack of an effect of SIRT6 depletion is consistent with the role of this sirtuin in poly [ADP-ribose] polymerase (PARP)-1-dependent BER (65, 66), and our findings that PARP-1 does not participate in cytokine or nitric oxide-dependent DNA damage (67). Much like SIRT2 and SIRT6, knockdown of the nuclear-localized SIRT7 isoform (54) did not modify nitric oxide-stimulated *Gadd45α* mRNA accumulation in *Sirt1*^*−/−*^ INS 832/13 cells (Fig. 5*F*).

While SIRT3 is primarily thought of as a mitochondrial localized member of the sirtuin family, it has been observed in the nucleus of cells (57, 58, 59) and we find that a small fraction of SIRT3 can be found in the nucleus of INS 832/13 cells (Fig. 6*B*). Consistent with nuclear localization, depletion of SIRT3 resulted in an inhibition of nitric oxide-stimulated *Gadd45α* expression in both wild-type (Fig. 6*E*) and *Sirt1*^*−/−*^ INS 832/13 cells (Fig. 6*H*) as well as dispersed rat islet cells (Fig. 6*K*). Further, we have previously shown that nitric oxide-derived oxidants inhibit the enzymatic activity of human recombinant SIRT1, SIRT2, and SIRT6, but not SIRT3 deacetylase activity (68, 69). To test the role of SIRT3 in β-cell DNA repair, we showed that the SIRT3 selective inhibitor LC-0296 attenuates nitric oxide-dependent *Gadd45α* mRNA accumulation, and prevents the repair of damaged DNA in INS 832/13 cells (Fig. 7). Consistent with an inhibition of DNA repair, INS 832/13 cells become sensitized to nitric oxide such that DEA/NO, which alone did not modify INS 832/13 cell viability, increases the lysis of these cells to 40% in the presence LC-0296 (Fig. 7). Overall, these findings support SIRT3 as the member of the sirtuin family of NAD^+^-dependent deacetylases that participates in the regulation of *Gadd45α* mRNA accumulation and the repair of damaged DNA in β-cells.

SIRT3 deacetylates and increases the stability of eight-oxoguanine-DNA glycosylase 1, a mitochondrial BER enzyme (70) and over-expression of SIRT3 increases the efficiency of BER in human fibroblasts (65). While the mechanisms of action are unknown, nitric oxide stimulates FoxO1 nuclear localization and FoxO1-dependent *Gadd45α* mRNA accumulation and DNA repair in INS832/13 cells (35). The FoxO family of transcription factors each have similar structures and functions (71), and SIRT3 can deacetylate FoxO3 (72, 73). Further, we have shown that acetylated FoxO1 accumulates in response to nitric oxide and EX-527 (35). Since FoxO1 is the predominant isoform found in islets (74, 75) and SIRT3 deacetylase activity is sensitive to EX-527, we hypothesize that the deacetylation of FoxO1 by SIRT3 is required for nitric oxide-stimulated *Gadd45α* mRNA expression and DNA repair in β-cells. In support of this hypothesis, we show that acetylated FoxO1 accumulates in INS 832/13 cells treated with the SIRT3 inhibitor LC-0296 and DEA/NO (Fig. 7*C*).

Exposure of islets to IL-1 results in nitric oxide-dependent damage to β-cell DNA (22, 23, 41, 61), which can activate the DDR as evidenced by increases in H2AX phosphorylation (referred to as γH2AX) (24). However, in a β-cell selective manner, nitric oxide is a potent inhibitor of DDR kinase signaling (ATM and ATR) (26, 76, 77) when produced at iNOS-derived low μM levels, or levels that inhibit mitochondrial oxidative metabolism. Because glycolysis and mitochondrial oxidative metabolism are coupled in β-cells, the net result of the actions of iNOS-derived levels of nitric oxide is a decrease in ATP to levels that fall below the K_m_ of glucokinase (30, 76). This limits glucose uptake and places the β-cells in a state of “metabolic suspended animation”, where glucose is transported across the membrane, but it is not readily phosphorylated because the levels of ATP fall far below the K_m_ for this substrate for glucokinase (30). This action is responsible for the inhibition of insulin secretion following cytokine treatment of islets and is the basis for the belief that cytokines participate in, or mediate, the loss of functional β-cell mass during diabetes development (5, 18). However, β-cells use this same mechanism to attenuate apoptosis (76), limit picornavirus replication (78, 79), and stimulate pathways that activate the repair of damaged DNA (34, 35), as we described in this study.

Recently, we have explored the concentration-dependent manner by which nitric oxide stimulates the expression of several genes that participate in the recovery of β-cells from cytokine-mediated damage (80). At concentrations below the levels that inhibit mitochondrial oxidation (0.5–1 μM), nitric oxide stimulates the expression of genes involved in DNA repair (*Gadd45α*), mitochondrial biogenesis (*Ppargc1α*), and pathways that alleviate cellular stress such as the unfolded protein (*Chop*) and heat shock (*Hsp70* and *Hmox1*) responses. As the concentrations increase to levels that inhibit oxidative metabolism and insulin secretion (1–2 μM), nitric oxide fails to stimulate the expression of these protective genes; however, at these iNOS-derived levels, nitric oxide is an effective inhibitor of apoptosis (25, 26, 76) and picornavirus replication in a β-cell selective manner (78, 79). These findings indicate that as nitric oxide levels increase in response to cytokine treatment, one of the first events is the increase in the expression of protective genes (80). As the concentrations of this free radical continue to increase, they reach levels that attenuate oxidative metabolism, gene expression, insulin secretion, and mitochondrial metabolism (33, 76). As nitric oxide concentrations fall below this inhibitory level of 1 to 2 μM (32, 80), mitochondrial oxidative metabolism recovers, insulin secretion resumes (32, 33) and damage to DNA is repaired (34). It is only when islets are exposed to cytokines for extended periods of 36 h or longer that all these actions become irreversible and cell death results (33). This irreversible damage is associated with the loss of NAD^+^/NADH, NADP^+^/NADPH, and ATP (30). Importantly, SIRT3 is an NAD^+^-dependent deacetylase that when active keeps FoxO1 deacetylated in the nucleus and results in the expression of genes involved in DNA repair (*e.g. Gadd45α*) (43, 44, 57). However, as NAD^+^ pools are depleted and SIRT3 is less active, FoxO1 is acetylated and switches to stimulate the expression of proapoptotic genes (*e.g. Puma* and *Noxa*) (43, 81). Future studies focused on identifying the mechanisms responsible for regulating these protective pathways, and the events that trigger irreversible inhibition of function will be essential in determining the physiological actions of cytokines on β-cells. In this article, we identify SIRT3 as a key deacetylase responsible for regulating FoxO1-dependent *Gadd45α* expression and DNA repair as one physiological role for IL-1 signaling in β-cells.

## Experimental procedures

### Materials

Nitric oxide donors (Z)-1-(N,N-Diethylamino)diazen-1-ium-1,2-diolate (DEA/NO), (Z)-1-[N-(3-aminopropyl)-N-(3-ammoniopropyl)amino]diazen-1-ium-1,2-diolate (DPTA/NO), and sirtuin inhibitors 6-chloro-2,3,4,9-tetrahydro-1H-carbazole-1-carboxamide (EX-527), and 2-[(4,6-dimethyl-2-pyrimidinyl)thio]-N-[5-(1-naphthalenylmethyl)-2-thiazolyl]-acetamide (SirReal2) were purchased from Cayman Chemical. Methyl N_2_-((benzyloxy)carbonyl)-N_5_-(1-(3-carbamoylbenzyl)-1H-indol-4-yl)-L-glutaminate (LC-0296) was purchased from Aobious (Gloucester, MA). Neutral red was purchased from Sigma Aldrich. Human recombinant IL-1β was purchased from Peprotech. *N*^*G*^-methyl-L-arginine (NMMA) was obtained from Enzo Life Sciences. INS 832/13 cells were a gift from Chris Newgard (Duke University). RPMI 1640 medium, Dulbecco’s modified eagle medium (DMEM), trypsin, and _L_-glutamine were purchased from Corning. Sodium pyruvate, HEPES, sodium nitrite, and β-mercaptoethanol were purchased from Thermo Fisher Scientific. Fetal bovine serum was procured from Hyclone Laboratories. The following antibodies were purchased from Cell Signaling Technology: rabbit anti-FoxO1 (9454, 2880), rabbit anti-AMPK (2603), mouse anti-SIRT1 (8469), rabbit anti-SIRT2 (12,672), rabbit anti-SIRT3 (5490), rabbit anti-SIRT6 (12,486), and rabbit anti-SIRT7 (5360). Mouse anti-α-Tubulin (GTX27291) antibody was obtained from GeneTex and mouse anti-GAPDH (AM4300) was purchased from Thermo Fisher. Rabbit anti-H3 (ab1791) and anti-MnSOD (ab13533) were purchased from Abcam and mouse anti-α-Tubulin was purchased from Sigma (T9026). Donkey anti-rabbit and anti-mouse secondary antibodies conjugated to horseradish peroxidase (HRP) were purchased from Jackson ImmunoResearch Laboratories.

### Cell culture and rodent islet isolation

INS 832/13 rat insulinoma cells were cultured in RPMI 1640, 10% FBS, 2 mM _L_-glutamine, 1 mM pyruvate, 10 mM HEPES, and 50 μM β-mercaptoethanol as previously described (26) and maintained in an atmosphere of 5% CO_2_ at 37 °C. Cells were removed from culture plates with 0.05% trypsin in 0.53 mM EDTA and plated at densities of 375,000 cells/ml (siRNA transfections) or 500,000 cells/ml (all other experiments). Islets from male Sprague Dawley rats were isolated as previously described (82) and cultured at 37 °C with 5% atmospheric CO_2_. Islets were dispersed into individual cells by trypsin digestion (83). The Institutional Animal Care and Use Committees at the Medical College of Wisconsin (A3102–01) have approved all animal care and procedures.

### FoxO1 adenoviral transduction

INS 832/13 cells were transduced with 50 pfu/cell of wild-type FoxO1 or dominant negative FoxO1 Δ*256* adenoviral constructs as previously described (35, 42). Following a 1 h incubation with the adenoviral constructs, the cells were washed and cultured for an additional 24 h before experiments were performed.

### Generation of Sirt1^−/−^ INS 832/13 cells

*Sirt1*^*−/−*^ INS 832/13 cells were generated as previously described (45) using two sgRNAs specific for rat *Sirt1*, (5′-GTCATCGTCATCACTTTCAC-3′) and (5′-CACACGCAAGCTCTAGTGAC-3′) that were subcloned into the pX462 Cas9n vector (Addgene #62987) using FastDigest *Bpi*I and T4 DNA ligase (Thermo Fisher) as previously described (84). The sgRNA vectors were co-transfected into INS 832/13 cells using Lipofectamine 2000 (Thermo Fisher). Transformants were selected with puromycin (1 μg/ml), and individual colonies were isolated for expansion after 10 to 14 days. Western blot analysis was used to screen for knockout cells, and sequencing was performed by Functional Biosciences. The sgRNAs produced a line of *Sirt1*^*−/−*^ INS 832/13 cells with early truncations in both gene copies (Δ*166*/Δ*167*).

### RNA sequencing

Total RNA was isolated from INS 832/13 cells and *Sirt1*^−/−^ INS 832/13 cells using a RNeasy kit (Qiagen). Genomic DNA was removed using a TURBO DNA-free kit (ThermoFisher Scientific). Library preparation and sequencing were completed by the Mellowes Center for Genomic Sciences and Precision Medicine Center (Medical College of Wisconsin). In brief, cDNA was synthesized from 5 to 10 ng of input RNA using the SMARTseq Ultra Low Input workflow (Takara Bio Inc) with libraries prepared and amplified with Nextera DNA Library kit (Illumina). Paired-end sequencing was performed on the Illumina NovaSeq6000. Reads were aligned to the reference genome (rn7.2) and differential expression analysis was performed using DE-seq (85).

### Sirtuin activity assays

Recombinant human SIRT1, SIRT2, SIRT3, SIRT5, and SIRT6 were expressed in BL21 (DE3) *E. coli* and purified by nickel-affinity chromatography as previously described (69, 86). Deacetylase activity for SIRT1-3 was determined using a continuous enzyme-coupled sirtuin assay (87). Activity was measured at 25 °C in buffer containing 20 mM potassium phosphate pH 7.5, 2 to 2.5 μM maltose-binding protein-tagged pyrazinamidase, 3.3 mM α-ketoglutarate, 200 μM NADH, 2.5 U _L_-glutamic dehydrogenase, and 100 μM NAD^+^ (SIRT2 and SIRT3) or 500 μM NAD^+^ (SIRT1), and 0.5 μM SIRT1-3 enzyme. The following acetylated peptide concentrations were used: 100 μM p53ac (SIRT1), 30 μM H3K14ac (SIRT2), or 100 μM ATPαac (SIRT3) (86). Assays were initiated by the addition of NAD^+^ and acetylated peptides. Activity was monitored using a FlexStation multimode microplate reader (Molecular Devices). Desuccinylase activity of SIRT5 and demyristoylase activity of SIRT6 were determined using an HPLC deacylase assay (88), which was performed at 25 °C in buffer containing 20 mM potassium phosphate pH 7.5, 20 μM NAD^+^ (SIRT5) or 31 μM NAD^+^ (SIRT6), 12.5 μM GDHsucc (SIRT5) or 20 μM H3K9myr (SIRT6), and 0.5 μM SIRT5 or SIRT6. Assays were initiated by the addition of NAD^+^ and acetylated peptide and quenched with 1% TFA after 2.5 min (SIRT5) or 45 to 60 min (SIRT6). Samples were centrifuged (15,000*g*) to remove debris, and peptides were resolved by HPLC (Agilent 1100) on a C18 column (4.6 × 250 mm, Thermo Fisher). An acetonitrile gradient (5–50%) in water with 0.1% TFA was applied over 20 min (SIRT5) to resolve GDH (13 min) and GDHsucc (14.4 min) peptides. An acetonitrile gradient of 5 to 70% in water with 0.1% TFA was applied over 40 min (SIRT6) to resolve H3K9 (15.3 min) and H3K9myr (21.5 min) peptides. The area under the curve at 230 nm (GDHsucc) and 280 nm (H3K9myr) was integrated to calculate the percent conversion of peptides. Sirtuin assay peptides were synthesized using solid-phase techniques (69).

### siRNA knockdown

Reverse transfection was performed on INS 832/13 or dispersed rat islet cells using Lipofectamine 2000 and Opti-MEM reduced serum media (Thermo Fisher) according to the manufacturer's instructions. Final siRNA concentrations used were 40 pmol/well. Media was changed 6 h after transfection (INS 832/13 and dispersed islet cells) and the cells were cultured 48 h before experiments were performed. Knockdown effectiveness was evaluated by RT qPCR and Western blot analysis. Negative Control (NC1) siRNA and dicer-substrate siRNAs targeting *Sirt2*, *Sirt3*, *Sirt6*, and *Sirt7* were purchased from Integrated DNA Technologies.

### Quantitative RT-PCR

RNA was isolated from whole cell lysates with the RNeasy kit (Qiagen). Samples were treated with Turbo DNA-free DNase (Thermo Fisher), and first-strand cDNA synthesis was performed using oligo(dT)_20_ primers (Integrated DNA Technologies) and Maxima H-Minus reverse transcriptase (Thermo Fisher). RT qPCR was performed using SsoFast EvaGreen supermix with the CFX96 Real Time System (Bio-Rad). Values were normalized to *Gapdh*, and relative fold change was calculated by the ΔΔCT method. Primers used for qPCR are shown in Table 1.

**Table 1:** qPCR Primers

Gene	Species	Primer	Sequence 5′-3′
*Gadd45*α	Rat	For	GTGTGCTGGTGACGAACCCACAT
		Rev	CCGTTCGGGGAATCACCGTCCG
*Gapdh*	Rat/Mouse	For	GACATCAAGAAGGTGGTGAAGC
		Rev	TCCAGGGTTTCTTACTCCTTGG
*Hsp70*	Rat	For	GCAACGTGCTCATCTTCGAC
		Rev	AAGTCCTCCCCGCCCA
*Sirt2*	Rat	For	ACTCGGACACTGAGGGAGG
		Rev	CCAGCTCCCACCAAACAGAT
*Sirt3*	Rat	For	GTGGCCTCTACAGCAACCTT
		Rev	GCTTTGAGGCAGGGATACCA
*Sirt6*	Rat	For	CTCGCCCCCAAGTTTGACAT
		Rev	GGGGCACTCCTCTACAAACA
*Sirt7*	Rat	For	CGAGTGTTTGACGTGACGGA
		Rev	TTCGTCATGCACCAGAGACG

### Immunoprecipitation and Western blot analysis

Acetylated lysine residues were immunoprecipitated from INS 832/13 cells using mouse anti-acetylated lysine antibody (Cell Signaling, Beverly, MA) coupled to Dynabeads M-270 Epoxy (Thermo Fisher) at a concentration of 20 antibody per μg magnetic bead, according to the manufacturer’s instructions. After treatment, INS 832/13 cells (∼1,000,000 cells per condition) were harvested in 120 μl RIPA buffer (Pierce) containing 1× protease inhibitor cocktail (Roche) and 90 μl of the lysate was mixed with 10 μl of acetylated lysine antibody-coupled magnetic beads and incubated overnight at 4 °C with end-over-end mixing. Beads were washed twice with 0.1% BSA/PBS and once with PBS, and samples were prepared for SDS-PAGE by the addition of 40 μl of Laemmli buffer.

Cells were washed with PBS pH 7.4, lysed in 1× Laemmli buffer, and proteins were resolved by SDS-PAGE and transferred onto the nitrocellulose membrane. The membrane was blocked with 3% milk/BSA in Tris-buffered saline, 0.1% Tween 20 for 1 h, and incubated with primary antibody overnight at 4 °C. The following primary antibodies were used at a 1:1000 dilution: FoxO1, AMPK, SIRT1, SIRT3, SIRT7, and MnSOD. Primary antibodies for SIRT2 and SIRT6 were used at a 1:500 dilution and Histone H3 at 1:3000. The dilutions for α-Tubulin and GAPDH primary antibodies were 1:2000 and 1:40,000, respectively. Donkey anti-mouse and anti-rabbit HRP-conjugated secondary antibodies were used at a dilution of 1:10,000. Protein bands were detected by chemiluminescence (89).

### Cell fractionation

Fractionation of INS 832/13 cells was completed using a modified REAP method (90). Briefly, cells were washed with ice-cold PBS pH 7.4, scraped, and pelleted by centrifugation (>10,000*g* for 10 s). The cell pellet was resuspended in 450 μl ice-cold NP-40 (0.1% v/v), and 150 μl cell suspension was saved as the whole-cell fraction. The lysate was centrifuged (>10,000*g* for 10 s), and 150 μl supernatant was saved as the cytoplasmic fraction. The remaining supernatant was removed, and the pellet containing nuclei was resuspended in 100 μl of ice-cold NP-40 (0.1% v/v) and saved as the nuclear fraction. Samples were flash-frozen in liquid nitrogen and stored at −80 °C until further processing. Whole-cell and nuclear fractions were subjected to sonication *via* a Bioruptor Plus (Diagenode) before additional centrifugation (>10,000*g* for 3 min at 4 °C) to remove cell debris. Samples were analyzed by Western blot analysis as stated previously.

### Cell viability assays

The neutral red uptake and the SYTOX green uptake assays were used to measure cell viability in INS 832/13 cells as previously described (22, 26). Cells, cultured on 24-well plates and treated as described in Figure Legends, were incubated at 37 °C for 1 h with neutral red dye (final concentration, 40 μg/ml). After media removal, cells were fixed with a 1% formaldehyde, 1% CaCl_2_ solution, and Neutral red was extracted with 200 μl of a solution containing 50% ethanol, and 1% acetic acid. Absorbance at 540 nm was measured and percent viability was determined relative to untreated cells. For SYTOX green uptake, INS 832/13 cells were incubated with 5 μM SYTOX green for 5 min at 37 °C. Fluorescence was measured at excitation/emission of 504/523 nm, and percent cell death was calculated based on cell permeabilization with 120 μM digitonin (100% death).

### Comet assay

DNA damage was determined using the comet assay as previously described (91). In brief, cells were harvested by cell scraping with a rubber policeman, resuspended in 0.6% low melting point agarose, and applied to Comet Slides (R&D Systems) which were allowed to solidify. Lysis was performed during an overnight incubation at 4 °C in buffer containing 2.5 M NaCl, 100 mM EDTA, 10 mM Tris (pH 10.0), 0.1% SDS, and 1% Triton X-100, followed by a 40 min incubation in alkaline unwinding solution (300 mM NaOH, 1 mM EDTA, pH > 13). DNA was separated by electrophoresis at 25 V, 300 mA for 40 min. Slides were rinsed twice with distilled water, allowed to dry, rinsed once with 70% ethanol, and then incubated at 37 °C for 30 min to dry. Slides were stained with 1× SYBR Gold (Thermo Fisher) and imaged by fluorescent microscopy. DNA damage in individual cells was quantified using the Comet Assay Software Project v1.2.2 (© 2001–2003 Krzysztof Konca). Comets were analyzed in a blinded manner assessing 25 to 50 cells per experimental condition. Results are expressed as mean tail moment and % tail DNA.

### Nitrite determination

The Griess assay was used to determine nitrite levels in cell culture by mixing 50 μl culture medium with 50 μl Griess reagent (92). Absorbance at 540 nm was measured, and nitrite levels were calculated based on a sodium nitrite standard curve.

### Statistical analysis

One-way ANOVA was used to examine experimental results. Statistical significance (minimum *p* < 0.05) was determined by Tukey's multiple comparisons *post hoc* test.

## Data availability

All data not contained in the manuscript will be shared upon request to John A. Corbett, Medical College of Wisconsin, jcorbett@mcw.edu.

## Conflict of interest

The authors declare that they have no conflicts of interest with the contents of this article.
